# Cognitive Behavioral Immersion for Depression: Randomized Controlled Trial Comparing Virtual Reality and Flat-Screen Delivery

**DOI:** 10.2196/92347

**Published:** 2026-07-15

**Authors:** Iony D Ezawa, Francisco N Ramos, Steven D Hollon, Gloria T Han, Noah Robinson

**Affiliations:** 1 Department of Psychology, University of Southern California, 3620 S. McClintock Ave., Los Angeles, CA, United States, 1 213 740 2203; 2 Department of Psychology, Vanderbilt University, Nashville, TN, United States; 3 Department of Anesthesiology, Vanderbilt University Medical Center, Nashville, TN, United States; 4 Department of Pediatrics, Vanderbilt University Medical Center, Nashville, TN, United States; 5 XRHealth, Boston, MA, United States

**Keywords:** cognitive behavioral immersion, virtual reality, depression, randomized controlled trial, metaverse

## Abstract

**Background:**

Depression is prevalent and debilitating. Although interventions exist, they are rarely delivered in accessible, scalable ways that retain their effectiveness. Cognitive behavioral immersion (CBI) is a coach-led cognitive behavioral skills program delivered in social virtual worlds that offers a potential solution.

**Objective:**

This parallel-group, web-based randomized controlled superiority trial compared CBI accessed via virtual reality headsets (CBI-VR) or flat-screen devices (CBI-FS) to a delayed access control.

**Methods:**

Inclusion criteria included a clinical level of depression symptoms, age ≥18 years, able and willing to give informed consent, access to a computer with an internet connection, and ability to speak and read English. Eligible participants were randomized using a random number generation script in a 1:1:1 ratio to conditions. CBI consisted of 8 weekly 1-hour groups led by coaches who taught cognitive behavioral skills. The intervention lasted 8 weeks; follow-up lasted 6 months. The primary outcome was depression symptoms; secondary outcomes were anxiety symptoms and quality of life. Recruitment and study procedures were conducted online. Outcomes were assessed through electronic self-report questionnaires. The study was unblinded. Hierarchical linear modeling was used to examine differences in rates of change among conditions. We explored the sense of presence as a potential mediator of intervention response.

**Results:**

Participants were recruited from February 2024 to January 2025; n=102 were randomized to each condition. Participants randomized to CBI-VR and CBI-FS attended an average of 5 intervention sessions. Primary analyses included all participants in the intent-to-treat sample that completed ≥2 outcome surveys to estimate within-person change (CBI-VR: n=98; CBI-FS: n=86; control: n=102). CBI-VR showed faster reductions in depressive and anxiety symptoms than either CBI-FS (depression: *β*=.21; 95% CI 0.02-0.40; *P*=.03 and anxiety: *β*=.20, 95% CI 0.03-0.38; *P*=.02) or the control (depression: *β*=.31, 95% CI 0.13-0.48; *P*<.001 and anxiety: *β*=.18, 95% CI 0.01-0.34; *P*=.03) across the 8-week intervention, with improvements largely maintained over the 6-month follow-up. CBI-VR also showed greater improvements in general quality of life (*β*=−1.02; 95% CI −1.63 to −0.40; *P*=.001) and psychological well-being (*β*=−1.01, 95% CI −1.44 to −0.59; *P*<.001) than the control from pre- to postintervention. The sense of physical presence in the environment was associated with CBI-VR’s effects on depression symptoms (*ab*=−0.85, 95% CI −1.71 to −0.15). No adverse effects occurred in any group.

**Conclusions:**

This study evaluated the efficacy of an innovative coach-led cognitive behavioral skills group delivered via VR. To our knowledge, our trial is the first to demonstrate that CBI delivered via VR is effective. These findings extend prior work on digital cognitive behavioral therapy by supporting CBI-VR as an effective and viable intervention package for depression and anxiety symptoms. These findings may help inform future research on suitable technology that can help bridge mental health care gaps.

## Introduction

### Background and Rationale

Major depression affects an estimated 332 million individuals, making it one of the most prevalent mental health disorders in the world [1]. Alarmingly, this figure has only risen in recent decades [2]. Beyond its widespread occurrence, depression consistently ranks as a principal cause of disability and ill health [3] and is associated with a heightened risk of suicide [4]. Fortunately, depression is a treatable disorder. Over the past several decades, numerous interventions have been developed and rigorously tested [5], with cognitive behavioral therapy (CBT) [6] standing out for its efficacy in both treating active depression [7] and preventing subsequent relapse [8]. CBT aims to treat depression by teaching clients cognitive and behavioral skills to challenge and correct negative, inaccurate thoughts and engage in more adaptive behaviors [6].

Despite effective treatments such as CBT, the global burden of depression continues to rise, a phenomenon termed the depression-treatment paradox [9,10]. This paradox is not due to a lack of effective treatments but rather to failures in their dissemination [9,10]. For many, evidence-based mental health care remains out of reach because of limited provider availability and substantial logistical and financial barriers to access high-quality care [11]. There is a critical need for interventions that are scalable and accessible without compromising their therapeutic integrity to overcome this gap. Two strategies hold particular promise in this regard: using trained lay counselors (“coaches”) to deliver structured CBT skills [12] and leveraging technology to extend their reach beyond traditional clinical settings [13,14].

In recent decades, there has been a growing use of lay counselors (also commonly referred to as coaches, peer support, or paraprofessionals) to provide cognitive behavioral coaching [15]. Numerous studies show that individuals without prior professional therapy training can be trained to effectively deliver CBT-based interventions [eg, 15-17]. This strategy highlights a path in which effective mental health care can be more easily disseminated to individuals who might not have access to mental health care professionals who are often difficult to access due to barriers such as geographic limitations, high costs, and provider shortages [15,16].

The advent of digital mental health interventions has also increased access to mental health interventions by allowing individuals to access mental health care from any internet-connected device and at any time. While stand-alone self-guided digital interventions (such as apps or websites) have outperformed control conditions in the treatment of depression, comprehensive meta-analyses have demonstrated that digital interventions are more efficacious when delivered with the accompaniment of lay counselors [eg, 18]. While cellphone apps and websites do not typically integrate lay counselors into the digital intervention itself, recent advances in technology offer a unique opportunity to integrate the strengths of lay counselors directly into networked 3D virtual worlds (eg, social virtual worlds or “the metaverse”). Unlike conventional digital platforms that rely on solitary and asynchronous interactions, social virtual worlds instantiate users (both participants and lay counselors) as virtual avatars, allowing them to “enter” digital environments and engage in real-time social interactions [19,20].

Moreover, these 3D digital environments can be accessed via any internet-connected device, ranging from traditional flat-screen devices such as smartphones, tablets, or computers to fully immersive virtual reality (VR) devices. Immersive VR may heighten one’s sense of “presence,” the subjective feeling of actually “being” in a virtual environment [21]. Presence can be further separated into two components: environmental presence, the sense of being physically present in the digital environment, and social presence, the sense that one is interacting with and connecting to others in the environment and that these interactions feel socially “real” [22,23]. The heightened sense of presence promoted by immersive devices is thought to increase engagement and the emotional impact of digital interventions. Prior work has focused primarily on using VR for asynchronous interventions and exposure-based interventions for anxiety and trauma-related disorders [eg, 24-26]. However, using VR to deliver synchronous interventions may have special potential in treating depression. Delivering CBT as a group-based, coach-led intervention in social virtual worlds using immersive VR technology may preserve the interpersonal and skill-building aspects that make traditional CBT effective while simultaneously enhancing accessibility and scalability. To our knowledge, no randomized controlled trial has compared the efficacy of delivering a mental health intervention in networked virtual worlds via a flat-screen device vs an immersive VR headset.

Cognitive behavioral immersion (CBI; XRHealth, Inc) is the first coach-led, synchronous CBT skills program that can be delivered in social virtual worlds on either VR headsets or flat-screen devices through the Innerworld application (developed by the company of the same name) [19]. This program involves an 8-session structure focused on teaching and encouraging the kind of CBT skill development that has been shown to predict cognitive change and symptom improvements among those receiving treatment for depression [27,28]. The program is conducted in a group format led by a trained coach to foster interpersonal factors such as group alliance and social support. These factors have been shown to be important in cultivating symptom improvement in therapeutic settings [29] but are often displaced in asynchronous or self-guided digital interventions. Preliminary findings supported CBI’s feasibility [19] and potential for reducing depressive symptoms [30]. Although no harms related to CBI have been reported in the preliminary work on CBI, qualitative interviews with participants who have used CBI described a learning curve associated with learning how to navigate the session scheduling logistics and VR headsets [19]. However, its efficacy has not been rigorously tested, nor is it known whether VR is superior to flat-screen access. In addition, a comparison with a delayed access control (DAC) group would allow for CBI-related improvement to be separated from unrelated symptom change over time, enabling a more rigorous estimate of treatment efficacy.

### Objectives

In this study, we conducted a 3-arm randomized controlled trial comparing CBI accessed via a VR headset (CBI-VR) vs CBI accessed via a flat-screen device (CBI-FS) and each to a DAC in a sample of participants experiencing elevated levels of depression symptoms to establish causality [31]. The primary aim of this trial was to evaluate the efficacy of CBI in reducing depressive symptoms across the intervention period and a 6-month follow-up period, with anxiety symptoms and quality of life examined as secondary clinical outcomes across the same time periods. The primary endpoint was postintervention (week 9). We hypothesized that participants randomized to CBI-VR would experience greater improvement over time on all 3 outcome indices compared with those in CBI-FS or DAC. A further exploratory aim was to examine whether environmental or social presence mediated the intervention effect on depression scores. Participant safety and adverse effects were also monitored throughout the study. Findings from this study will contribute to the field’s understanding of how delivery modality influences outcomes in digital interventions delivered in social virtual worlds. This study will also provide broader information on whether using novel technologies translates into meaningful clinical benefits.

## Methods

### Patient and Public Involvement

No patients or members of the public were involved in the study design, conduct, or reporting of the trial.

### Trial Design

This study was a parallel-group, unblinded, phase 2, 3-arm randomized controlled superiority trial (registration date May 15, 2024; first participant enrollment: February 26, 2024). Individual participants were randomized equally (1:1:1) across 3 study arms.

### Changes to Trial Protocol

No changes were made to the protocol after the study commenced. Two exploratory cross-sectional mediation analyses were conducted to further explore findings from the primary analyses and are described in the Statistical Methods subheading below.

### Trial Setting

The trial was conducted entirely online, and study procedures were led by researchers at the University of Southern California (USC) in Los Angeles, California, United States. Participants were recruited online from across the United States. This was a single-site trial via USC; intakes and onboarding were delivered remotely through Zoom (Zoom Communications, Inc), and intervention sessions were delivered remotely through the Innerworld platform.

### Eligibility Criteria

Inclusion criteria for the study included (1) scoring ≥10 on the Patient Health Questionnaire-9 (PHQ-9), a cutoff commonly used to screen for major depressive disorder [32]; (2) aged ≥18 years; (3) able and willing to give informed consent; (4) having access to a computer with a stable internet connection to access the CBI app; and (5) able to speak and read English. The exclusion criterion for this study was the presence of elevated suicide risk during the baseline assessment necessitating a higher level of care. Computer or internet literacy was not a de facto eligibility criterion. Participants self-selected to be in the trial and were recruited through posts on clinical trial registries (eg, ClinicalTrials.gov), advertisements on social media apps (ie, Facebook and Instagram [Meta Platforms, Inc]), investigators’ laboratory websites, and word of mouth. No strict eligibility criteria were used to select coaches. Instead, coaches were selected by the Innerworld staff based on interviews assessing responses to a variety of simulated scenarios that would be similar to what they might encounter in Innerworld. Selected coaches completed standardized training (further details provided in the Intervention and Comparator subheading below) before independently delivering the intervention in the trial.

### Intervention and Comparator

Interested participants completed a prescreening questionnaire on REDCap (Vanderbilt University) [33] containing a description of the study, eligibility questions, and the PHQ-9. Participants who met initial inclusion criteria were then invited to schedule a 20-minute intake video session conducted by study personnel to confirm eligibility and explain study procedures. The completion of baseline measures was also confirmed during intake sessions. Participants who consented to the study and whose eligibility was confirmed during the intake were subsequently randomly assigned in a 1:1:1 ratio to CBI-VR, CBI-FS, or DAC. All conditions lasted 8 weeks, with a 6-month follow-up period. Participants in all conditions were sent electronic self-report surveys to complete once a week during the intervention period, once at postintervention, and once a month during the follow-up period.

Participants assigned to the CBI-VR and CBI-FS conditions were emailed information regarding their randomization and instructions on how to download and use the application that delivered the intervention. Those in the CBI-VR group were mailed a Meta Quest 3 (Meta Platforms, Inc) headset before their first session, while participants in the CBI-FS group were instructed to use their own flat-screen device to access the intervention. Upon signing in to the application for the first time, participants were required to complete a web-based tutorial about the application. Additionally, they were offered the option to join a live onboarding session with study staff for additional support before their first session. Participants assigned to the DAC condition were emailed information about their randomization and were not told specifically about Innerworld or the CBI intervention until the postintervention period, at which point they were provided with detailed information explaining how to access the platform and intervention. Participants in all conditions were not prohibited from accessing concomitant care during the trial. Concomitant care was not measured systematically in this trial.

The CBI intervention was composed of 8 weekly, 1-hour intervention sessions hosted on the Innerworld application (Innerworld; developed by the company of the same name). Sessions were led by lay counselors (“coaches”) who had completed a structured 20-hour training program devised by Innerworld staff and who attended weekly supervision led by Innerworld support staff and study investigators to support fidelity to the intervention protocol. CBI sessions followed a CBT-informed intervention manual and accompanying checklists [6]. Typical sessions included agenda setting, reviewing homework assigned from the previous session, teaching and practicing new CBT skills, summarizing new content, assigning homework, and gathering feedback about the session. Groups were planned to contain up to 10 participants. To support participant adherence to sessions, participants could sign up for electronic session reminders, and participants unable to attend their assigned weekly session were encouraged by email to attend makeup sessions offered on a rotating schedule. Technical support was available through in-app staff avatars and a technical help desk. Although the intervention protocol itself was not modified during the trial, predefined safety-monitoring procedures guided additional assessment and consideration of intervention discontinuation if clinically indicated (refer to the Harms subheading below).

### Outcomes

Depression symptoms were measured as the primary outcome because CBI was designed to be a CBT-based intervention for depression. Anxiety symptoms and quality of life were measured as secondary outcomes to assess other clinical and functional changes associated with the intervention. These outcomes are not part of a core outcome set. Depression symptoms were assessed using the PHQ-9, a 9-item self-report measure of depression symptoms [34]; scores range from 0 to 27. Anxiety symptoms were assessed using the Generalized Anxiety Disorder-7 (GAD-7) [35]; scores range from 0 to 21. Quality of life was assessed using the World Health Organization Quality of Life Brief (WHOQOL-BREF) measure [36]; this measure contains 6 subscales assessing general quality of life (1 item), general perception of health (1 item), physical health (7 items), psychological health (6 items), social relationships (3 items), and environmental health (8 items). All WHOQOL-BREF subscales were standardized to range from 0 to 100. We used a 3-item measure to assess social presence (ie, how socially connected participants felt with others in the Innerworld platform), adapting the language to assess the participant’s experience inside the intervention application [22]; scores ranged from 3 to 21. We used a 5-item measure to assess environmental presence (ie, the degree to which the participant felt physically immersed in the Innerworld platform), adapting the language to assess the participant’s experience inside the intervention application [23]; scores ranged from 5 to 25. We used the 4-item Group Session Rating Scale (GSRS) as a proxy for session quality [37]; scores ranged from 0 to 40. All measures have been used online in previous studies. All outcomes were assessed using self-report. While postintervention (week 9) was designated as the primary endpoint for between-group comparisons, the main analysis metric for each primary and secondary outcome was the trajectory of change over time. Therefore, these outcomes were assessed at multiple points throughout and after the intervention period. Specifically, all participants completed the PHQ-9 and GAD-7 at baseline, once per week during the 8-week intervention (or delay) phase, at postintervention (week 9), and once per month during the 6-month follow-up period. The WHOQOL-BREF was assessed at baseline and postintervention. Participants randomized to the CBI-VR and CBI-FS conditions completed the presence measures at postintervention and the GSRS weekly during the 8-week intervention. No qualitative feedback was systematically obtained from participants.

### Harms

Participant safety was closely monitored. Coaches and investigators completed crisis management training and observed participants during CBI sessions for any indication of safety concerns. In addition, automated REDCap survey alerts were used to systematically identify elevated suicide risk and clinically significant symptom worsening during weekly assessments across the intervention period. Suicide risk was assessed using the PHQ-9, with scores ≥2 on the PHQ-9 suicide item triggering provision of crisis resources and administration of the Columbia-Suicide Severity Rating Scale (C-SSRS) self-report [38]. Those scoring ≥4 on the C-SSRS self-report were contacted by the study team to schedule a C-SSRS interview over Zoom. Symptom worsening alerts were triggered by ≥20% week-to-week increases in PHQ-9 or GAD-7 scores when total scores were ≥15, prompting contact and resource provision via email by the study team. C-SSRS interviews were conducted by research personnel at USC under the supervision of a licensed clinical psychologist. Personnel conducting C-SSRS interviews and safety-monitoring procedures were unblinded to study conditions. Harms and safety concerns were monitored continuously throughout participant enrollment and intervention participation.

No deaths or serious adverse events occurred in any study condition. No participants were withdrawn due to harms or safety concerns. One participant in the VR condition required C-SSRS interviews (twice) after enrolling in the study; however, this participant was determined not to be at immediate risk during both interviews and was subsequently provided with additional crisis resources and monitored closely throughout the remainder of the study. No safety concerns were reported by coaches from observed interactions in the intervention platform. Additionally, no critical secular events regarding computer hardware, internet resources, or privacy breaches occurred.

### Sample Size

We recruited 306 participants (n=102 per arm) based on an a priori longitudinal power analysis using the *powerlmm* package in R for the primary outcome of depression symptoms. We targeted a medium effect size (*d*=0.50) at post intervention (an effect size that is consistent with expectations for interventions targeting depressive symptoms) with >80% power and *α*=.05 (2-tailed) in a 2-level random coefficients hierarchical linear model (HLM), comparing 2 active conditions and taking into account an expected attrition rate of 25% based on typical rates of dropout in digital CBT treatment (refer to published protocol [31] for full details of the power analysis). No interim analysis was conducted. In addition, no stopping guidelines were prespecified; however, study investigators met with an independent data monitoring committee throughout the duration of the trial in the event of any safety or unexpected concerns that would warrant consideration of stopping the trial.

### Randomization

Random assignment was conducted by the lead research assistant at USC using a random number generation script executed in Python. Participants were randomized in a fixed 1:1:1 ratio to conditions using blocked randomization to distribute participants equally across conditions within each recruitment wave and across the trial duration (n=102 per arm). Recruitment occurred in waves based on coach and researcher availability. Block sizes were variable and corresponded to recruitment wave size, ranging from 24 to 55 participants. No stratification was conducted. The lead research assistant and principal investigator became aware of the block size for a given wave only after that recruitment wave was completed. The randomization script and procedure were prepared before participant enrollment, but the allocation sequence for a given recruitment wave was generated only after all eligible consenting participants in that recruitment wave completed baseline procedures to prevent allocation from influencing eligibility determination, consent, or baseline procedures. The allocation script and allocation sequence for each wave were securely stored in an approved university drive accessible only to the lead research assistant and the principal investigator at USC. The lead research assistant generated the allocation sequence and implemented assignment to study conditions. Other research assistants conducted eligibility evaluation, informed consent, and baseline procedures, but did not have access to the allocation sequence.

### Blinding

All study participants, coaches, and research personnel (data collectors and analysts) were unblinded because the conditions were unavoidably distinct and easily identifiable due to the device type used to access the intervention.

### Statistical Methods

HLM, a type of mixed effects regression that accounts for repeated measurements nested within individuals, was prespecified to examine our clinical outcomes. This approach allows for the modeling of individual trajectories of change over time while estimating average trends across intervention conditions. Fixed effects captured overall group patterns, whereas random effects accounted for individual differences in intercepts and slopes. Random intercepts and slopes were allowed to covary freely and were specified using an unstructured covariance matrix, which places no constraints on their variances or covariances. Models were estimated using restricted maximum likelihood, with degrees of freedom computed using the Kenward-Roger approximation to improve small-sample inference and control type I error.

Separate prespecified models with identical specifications were estimated for each outcome variable (PHQ-9 as the primary outcome, GAD-7 and WHOQOL-BREF subscales as secondary outcomes). Time was modeled using a piecewise linear specification to estimate distinct trajectories during the intervention and follow-up periods, with baseline outcome scores included as covariates. Two time variables were defined: (1) weeks since baseline during the intervention period (coded as 0 during follow-up) and (2) weeks since the start of the follow-up period (coded as 0 during the intervention). Accordingly, time was separated by creating 2 variables, 1 that represented weeks since baseline during the intervention period and coded as 0 during follow-up, and a second variable representing weeks since the start of the follow-up period and coded as 0 during the intervention. Fixed effects included condition (CBI-VR, CBI-FS, and DAC), both piecewise time variables, and their interactions with condition (time × condition), allowing estimation and comparison of condition-specific linear slopes for each period. Random intercepts and random slopes for both piecewise time variables were specified at the participant level. To further examine when group differences emerged, a second set of models treated time as categorical. In these models, random slopes were removed and random intercepts were retained. Least squares means were estimated for each condition at each week, with pairwise comparisons and slice tests used to identify specific timepoints showing significant differences. For the models predicting WHOQOL-BREF score, fixed effects for baseline scores and random slopes for time were not included because this variable was measured at only 2 timepoints, and including these terms prevented model convergence. All HLM analyses included all participants in the intent-to-treat sample that completed at least 2 outcome surveys to estimate within-person change (CBI-VR: n=98; CBI-FS: n=86; control: n=102); the remaining 20 participants who did not meet this criterion were excluded from primary analyses given that within-person change could not be estimated for these participants. We also descriptively reported the occurrence of adverse events by condition; between-group comparisons were not conducted due to the absence of adverse events. Analyses were conducted using SAS (version 9.4; SAS Institute Inc) with the MIXED procedure [39]. No adjustments for multiple comparisons were applied.

Exploratory analyses were conducted to examine whether environmental or social presence at post intervention mediated the effect of condition (CBI-VR vs CBI-FS) on postintervention depressive symptoms, controlling for baseline depression symptoms. To test whether participants’ sense of presence statistically mediated intervention effects, we used the PROCESS macro for SAS [40]. We used Model 4 with 1000 bootstrap samples to generate 95% CIs; CIs that did not contain 0 were interpreted as statistically significant.

Missing data for repeated outcomes were handled using restricted maximum likelihood–based estimation in the HLMs. No imputation was used. Little’s Missing Completely At Random test was used post hoc to evaluate whether data were consistent with missing completely at random, implemented in the *BaylorEdPsych* package in R (R Development Core Team). In sensitivity analyses, we evaluated a pattern-mixture model that extended the primary HLM to account for potential nonrandom missingness by examining models stratified on plausible missing data patterns (eg, dropout status).

Finally, we conducted post hoc analyses to examine the potential confounding role of CBI coaches. We first evaluated whether session quality (GSRS scores) varied by coach using a linear mixed effects model with participant as a random effect. We then reestimated the primary HLM predicting depression symptoms with coach included as a covariate. Additional ancillary analyses examining intervention change processes are beyond the scope of the present report and will be published separately.

### Ethical Considerations

The protocol for this trial was approved by the USC Institutional Review Board (UP-23‐00491). Study procedures were overseen by an independent Data and Safety Monitoring Board. Study data were stored in REDCap [33], a secure, university-approved platform for research study databases. All study data were deidentified before analysis. No individual participants or users are identifiable in any images included in the paper or additional materials. All participants provided written informed consent before beginning the study. Consent was obtained by having participants read the relevant forms, followed by a study meeting in which personnel explained the procedures and answered any remaining questions. Participants then signed and dated the form. All participants in each condition were compensated with US $10 for each survey completed as well as with a personal VR headset after completing their 8-week acute condition (CBI-VR participants kept the headset used during the intervention period). This study was reported in accordance with the CONSORT-EHEALTH (Consolidated Standards of Reporting Trials of Electronic and Mobile Health Applications and Online Telehealth) guidelines for randomized trials of web-based interventions [41] and the CONSORT (Consolidated Standards of Reporting Trials) 2025 expanded checklist and extension for abstracts checklist [42] (Checklists1  2).

## Results

### Participant Flow

**Figure 1.**
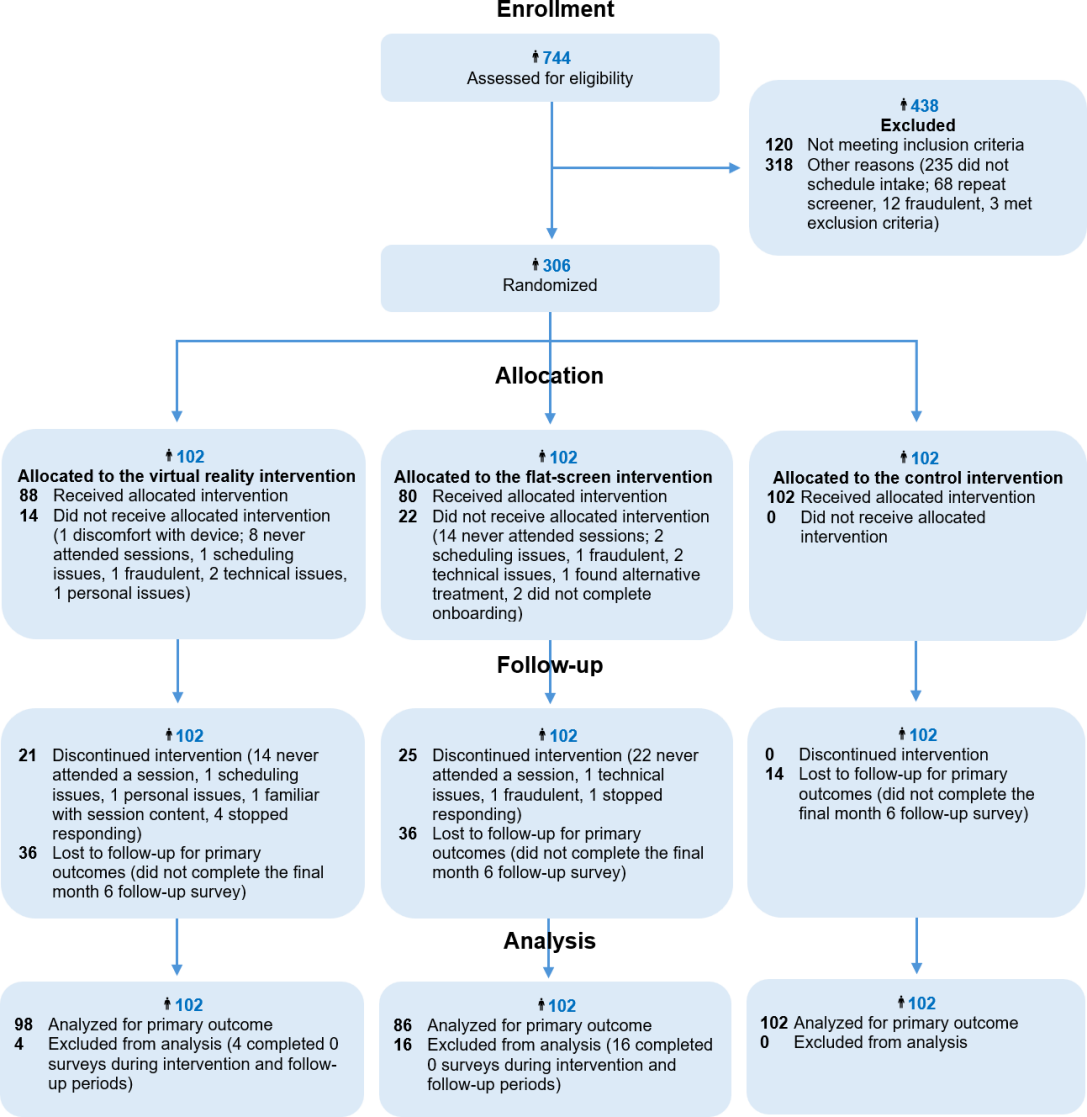
Flow diagram of participants through the study.

Figure 1 illustrates the participant flow through the study. A total of 744 individuals completed the initial eligibility screener. Of these, 306 individuals (whose eligibility was confirmed and who provided consent during a subsequent intake session) were randomized in a 1:1:1 ratio (CBI-VR: n=102, CBI-FS: n=102, DAC: n=102).

### Recruitment

Participants were recruited from February 2024 to January 2025. The first intervention groups (8 weeks in duration) began in March 2024, and the final groups ended in March 2025 when the prespecified sample size was reached. Follow-up data (6 months in duration) were collected from May 2024 to September 2025.

### Intervention and Comparator Delivery

The intervention was delivered by 8 peer coaches trained and staffed by Innerworld. Coaches received structured training, followed standardized manuals and session checklists, and participated in weekly supervision to support fidelity to the intervention protocol; however, coaches did not receive systematic observer-rated fidelity checks. CBI-VR and CBI-FS participants received the same 8-session CBI program through the Innerworld platform, accessed via VR headset and flat-screen device, respectively. DAC participants did not receive CBI during the 8-week intervention period but completed the same scheduled primary and secondary outcome assessments as the active conditions. Surveys were administered for all conditions by research personnel at USC. Participants in the CBI conditions completed an average of approximately 5 sessions (CBI-VR: mean 5.45, SD 2.95; CBI-FS: mean 5.39, SD 3.16). No significant differences were observed between the 2 CBI conditions in the number of sessions attended (CBI-VR: mean 5.45, SD 2.95; CBI-FS: mean 5.39, SD 3.16; *t*_202_=0.14; *P*=.89). All conditions showed large within-group improvements (*d*=0.92‐0.98). However, CBI-VR showed greater depressive symptom improvement than CBI-FS (*d*=0.23) and DAC (*d*=0.26). Similarly, all conditions demonstrated moderate to large improvements in anxiety (*d*=0.64‐0.79); CBI-VR showed a slightly greater improvement than CBI-FS (*d*=0.25) and DAC (*d*=0.18). While changes to external mental health treatment were collected via an optional open-response item, responses were not systematically coded.

### Baseline Data

Table 1 contains descriptive characteristics of the sample collected at baseline by study condition. Although there was considerable variability within each characteristic, the modal participant was a single White woman in her late thirties with moderate to severe levels of depression and anxiety and a middling quality of life. Results of one-way ANOVAs indicated no significant differences in symptoms or quality of life among the groups at baseline (depression: *P=*.37, anxiety: *P*=.96, general quality of life: *P*=.39).

**Table 1.** Baseline characteristics collected at intake for a randomized controlled trial of cognitive behavioral immersion (CBI) among adults with elevated depressive symptoms in the United States. CBI accessed via a virtual reality headset (CBI-VR) or a flat-screen device (CBI-FS) and compared with a delayed access control (DAC).

Variable		CBI-VR^a^ (n=102)^b^	CBI-FS^c^ (n=102)	DAC^d^ (n=102)
Age, mean (SD)		37.39 (12.10)	37.65 (13.38)	36.90 (11.67)
Sex, n (%)				
Female		71 (70)	64 (63)	74 (73)
Male		31 (30)	37 (36)	28 (27)
Not reported		0 (0)	1 (1)	0 (0)
Race and ethnicity^e^, n (%)				
Asian		12 (12)	27 (26)	23 (23)
Black		26 (25)	19 (19)	28 (27)
Hispanic		19 (19)	27 (26)	16 (16)
Native American		2 (2)	7 (7)	6 (6)
Pacific Islander		1 (1)	3 (3)	1 (1)
White		59 (58)	51 (50)	46 (45)
Other		3 (3)	2 (2)	6 (6)
Marital status, n (%)				
Single		63 (62)	60 (59)	61 (60)
Married		24 (24)	24 (24)	29 (28)
Widowed		5 (5)	1 (1)	1 (1)
Divorced		8 (8)	13 (13)	5 (5)
Separated		2 (2)	2 (2)	1 (1)
Not reported		0 (0)	2 (2)	5 (5)
Education, n (%)				
High school degree		12 (12)	5 (5)	9 (9)
Some college		19 (19)	25 (25)	19 (19)
Associate’s degree		14 (14)	7 (7)	10 (10)
Bachelor’s degree		39 (38)	45 (44)	40 (39)
Graduate degree		18 (18)	20 (20)	22 (22)
Not reported		0 (0)	0 (0)	2 (2)
Income, n (%)				
<US $20,000		11 (11)	9 (9)	10 (10)
US $20,000-US $49,999		26 (25)	26 (25)	19 (19)
US $50,000-US $74,999		14 (14)	20 (20)	17 (17)
US $75,000-US $99,999		17 (17)	15 (15)	15 (15)
US $100,000-US $149,999		15 (15)	13 (13)	17 (17)
US $150,000-US $199,999		7 (7)	6 (6)	8 (8)
US $200,000-US $249,999		3 (3)	3 (3)	1 (1)
US $250,000-US $299,999		1 (1)	2 (2)	1 (1)
≥US $300,000		0 (0)	2 (2)	2 (2)
Not reported		8 (8)	6 (6)	12 (12)
Patient Health Questionnaire-9 at intake, mean (SD)		16.86 (5.00)	16.27 (4.36)	15.93 (4.96)
Generalized Anxiety Disorder-7 at intake, mean (SD)		13.10 (4.85)	12.98 (4.86)	13.18 (5.06)
World Health Organization Quality of Life Brief measure at intake, mean (SD)				
General quality of life		51.96 (19.49)	55.88 (22.02)	52.94 (22.05)
General health		42.40 (23.01)	42.65 (22.41)	43.14 (24.67)
Physical health		54.12 (16.54)	54.76 (15.34)	53.74 (16.52)
Psychological health		35.16 (12.75)	37.54 (12.77)	37.97 (13.29)
Social relationships		43.10 (21.40)	44.36 (18.93)	43.18 (23.94)
Environmental health		59.53 (16.81)	61.34 (16.85)	59.61 (17.76)

a CBI: cognitive behavioral immersion.

b CBI-VR: CBI accessed via a virtual reality headset.

c CBI-FS: CBI accessed via a flat-screen device.

d DAC: delayed access control.

e More than one race or ethnicity could be selected.

### Numbers Analyzed, Outcomes, and Estimation

A total of 220 of 306 (71.9%) participants (CBI-VR: n=66, CBI-FS: n=66, and DAC: n=88) completed the final outcomes survey. Among this subset of participants, depression, anxiety, and quality-of-life subscale scores significantly improved over time. Consistent with the prespecified intent-to-treat analysis, 286 of 306 (93.5%) participants (CBI-VR: n=98, CBI-FS: n=86, and DAC: n=102) completed at least 2 outcome surveys and were therefore included in the primary and secondary analyses. At the survey level, approximately 18% (881/4896) of surveys were not completed. Refer to Multimedia Appendix 1 for a detailed table reporting the number of surveys missing per assessment point by condition. Sensitivity analyses further exploring patterns of missingness are reported in the Ancillary Analyses subheading below.

### Primary Analyses: Intervention Effects on Depression Symptoms Over Time

PHQ-9 scores were assessed at baseline, weekly across the 8-week intervention period and at post intervention, and monthly across the 6-month follow-up. An HLM controlling for baseline PHQ-9 scores examined PHQ-9 scores over time across the 3 conditions. PHQ-9 scores changed significantly during the intervention period (*F*_1,259_=229.31; *P*<.001), with rates of symptom reduction differing by condition (time × condition interaction: *F*_2,259_=6.03; *P*=.003). CBI-VR participants improved at a faster rate than either CBI-FS (*β*=.21, 95% CI 0.02-0.40; SE=0.09; *P*=.03) or DAC (*β*=.31, 95% CI 0.13-0.48; SE=0.09; *P*<.001); no difference was observed between the latter 2 conditions (*β*=.09, 95% CI −0.08 to 0.27; SE=0.09; *P*=.30). In other words, participants in CBI-VR exhibited greater reductions in PHQ-9 scores, decreasing by 0.21 more points per week than those in CBI-FS and by 0.31 more points per week than those in DAC, corresponding to approximately 1.9- and 2.8-point differences over the intervention period. Scores remained relatively stable over time across the follow-up period (*F*_1,243_=0.27; *P*=.60), although there was a nonsignificant time × condition interaction (*F*_2,243_=2.88; *P*=.06), such that participants in the CBI-VR condition showed a slightly higher increase in symptoms over time than CBI-FS (*β*=−.06, 95% CI −0.13 to −0.001; SE=0.03; *P*=.04) and DAC (*β*=−.07, 95% CI −0.13 to −0.01; SE=0.03; *P*<.03).

**Figure 2.**
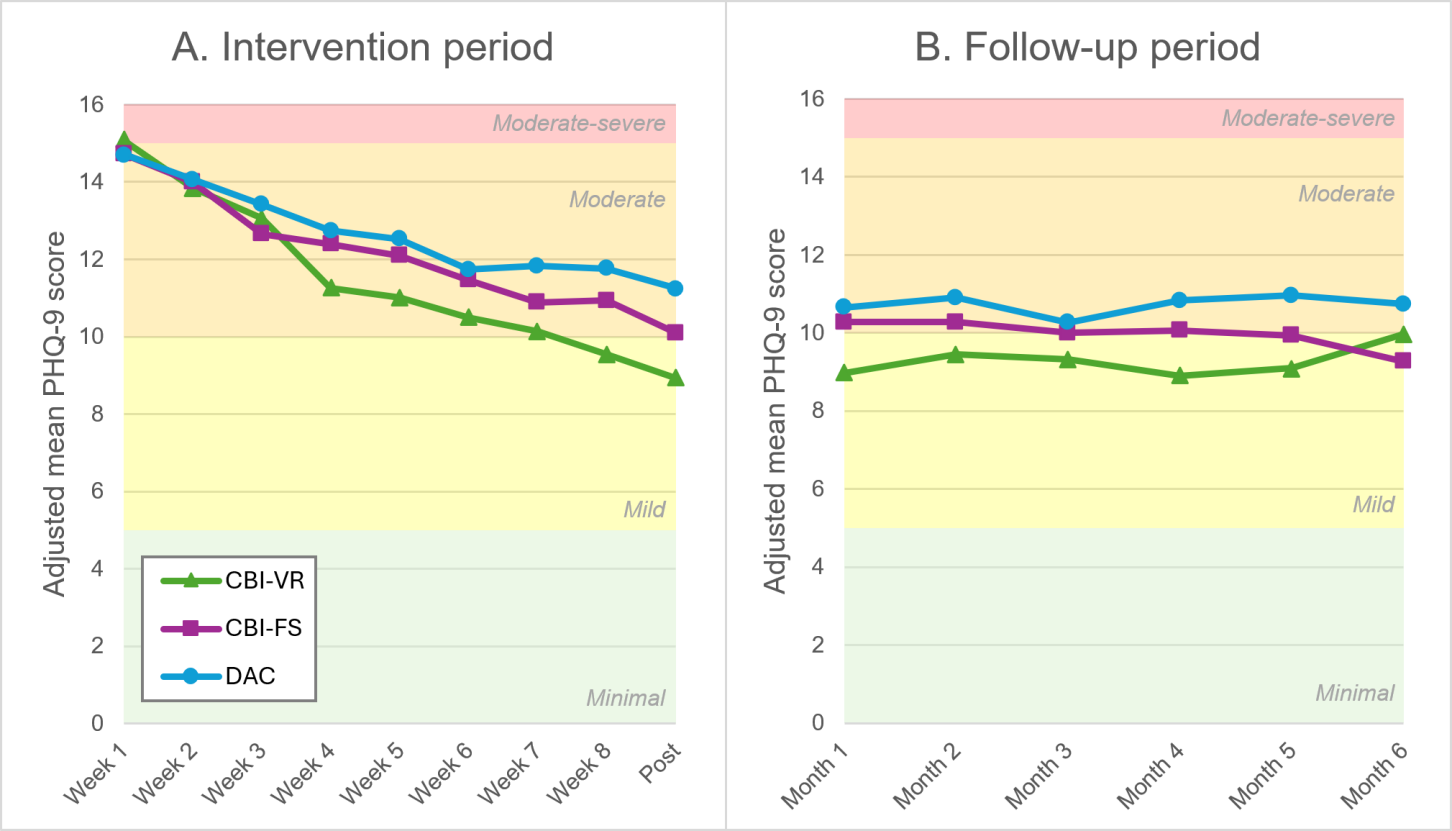
Adjusted mean depression scores over time by condition during the intervention and follow-up periods in a randomized controlled trial of cognitive behavioral immersion (CBI) among adults with elevated depression symptoms in the United States. CBI accessed via a virtual reality headset (CBI-VR) or a flat-screen device (CBI-FS) and compared with a delayed access control (DAC). Scores are least squares means from a mixed effects model adjusting for baseline depression and time (piecewise acute and follow-up phases). PHQ-9: Patient Health Questionnaire-9.

An HLM treating time as a categorical variable compared PHQ-9 scores across conditions at each timepoint. During the intervention period, no differences were observed in early weeks (weeks 1‐6; range of *Ps=*.11 to .95), but a nonsignificant trend emerged at week 7 (*P*=.08) that became fully significant by weeks 8 and 9 (both *Ps*=.01), favoring faster symptom reduction in CBI-VR over CBI-FS and DAC. PHQ-9 scores remained broadly stable across the 6-month follow-up period. In the VR condition, there was a small increase in PHQ-9 scores at the 6-month follow-up assessment relative to the postintervention assessment; however, scores at this timepoint remained numerically lower than those in the DAC condition and were not significantly different from those in the CBI-FS condition (*P*=.39). Examination of individual participant trajectories in the CBI-VR condition (n=59) indicated that this pattern was largely driven by minor within-person fluctuations and differential attrition rather than widespread symptom recurrence. Although 25 participants showed some increase in symptoms between the month 5 and 6 assessments, the majority of changes were small in magnitude, with only 3 participants’ symptoms crossing the relapse threshold (PHQ-9≥10) for the first time at the 6-month assessment. In addition, 5 participants with PHQ-9 scores ≥10 at month 5 did not complete the final assessment, contributing to a modest inflation of mean scores at follow-up. Figure 2 displays estimated least squares mean trajectories, with slice tests and pairwise comparisons reported in Multimedia Appendix 1.

While our primary analysis concerned continuous outcomes, we also examined response (defined as ≥50% reduction in postintervention PHQ-9 scores) and remission (defined as postintervention PHQ-9 ≤5) across the 3 conditions. Response rates were 48% (49/102), 33.3% (34/102), and 34.3% (35/102) for CBI-VR, CBI-FS, and DAC, respectively. A chi-square test comparing all 3 groups indicated a nonsignificant trend favoring CBI-VR (*χ²*_2_=5.82; *P*=.054, Cramér V=0.14). When comparing CBI-VR to DAC, the absolute risk difference was 13.73 percentage points (95% CI 0.35%-27.10%), and the relative risk was 1.40 (95% CI 1.00-1.96; *χ²*_1_=3.97; *P*=.046). Remission rates were 24.5% (25/102), 13.7% (14/102), and 19.6% (20/102) across the same conditions. A chi-square test comparing all 3 groups indicated findings in the same direction, but they were not significant (*χ²*_2_=3.82; *P*=.15; Cramér V=0.11). When comparing CBI-VR to DAC, the absolute risk difference was 4.90 percentage points (95% CI −6.46% to 16.26%), and the relative risk was 1.25 (95% CI 0.74-2.10; *χ²*_1_=0.71; *P*=.40).

### Secondary Analyses

#### Intervention Effects on Anxiety Symptoms Over Time

GAD-7 scores were also assessed at baseline, weekly across the 8-week intervention period and at postintervention, and monthly across the 6-month follow-up period. In an HLM controlling for baseline GAD-7 scores, scores changed significantly during the intervention period (*F*_1,260_=127.09; *P*<.001), with rates of symptom reduction differing by condition (time × condition interaction: *F*_2,260_=3.26; *P*=.04). CBI-VR participants improved faster than those in either CBI-FS (*β*=.20, 95% CI 0.03-0.38; SE=0.09; *P*=.02) or DAC (*β*=.18, 95% CI 0.01-0.34; SE=0.08; *P*=.03), with no differences between the latter 2 groups (*β*=−.02, 95% CI −0.19 to 0.14; SE=0.08; *P*=.77). In other words, GAD-7 scores in the CBI-VR condition decreased by approximately 0.20 more points per week than those in CBI-FS and by 0.18 more points per week than those in DAC, corresponding to approximately 2.0- and 1.8-point differences over the intervention period. Across the follow-up period, scores remained stable over time across conditions, with neither the main effect of time (*F*_1,243_=0.06; *P*=.80) nor the time × condition interaction (*F*_2,242_=0.27; *P*=.77) reaching significance. More specifically, participants in the CBI-VR condition did not show a difference in slope of symptoms as compared to CBI-FS (*β*=−.02, 95% CI −0.08 to 0.04; SE=0.03; *P*=.50) or DAC (*β*=−.003, 95% CI −0.06 to 0.05; SE=0.03; *P*=.91).

**Figure 3.**
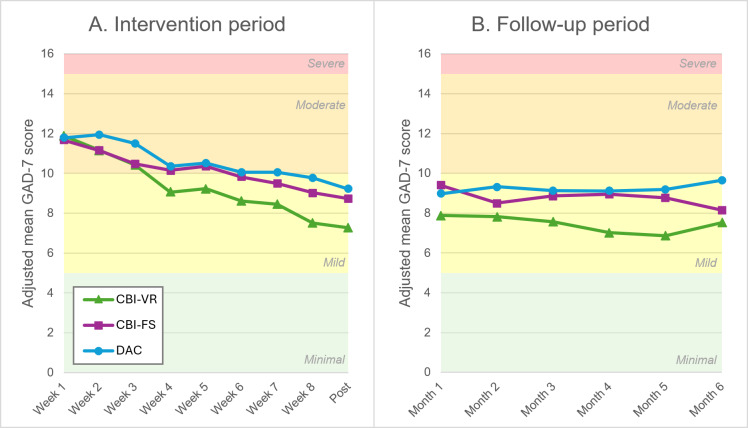
Adjusted mean anxiety scores over time by condition during the intervention and follow-up periods in a randomized controlled trial of cognitive behavioral immersion (CBI) among adults with elevated depression symptoms in the United States. CBI was accessed via a virtual reality headset (CBI-VR) or a flat-screen device (CBI-FS) and compared with a delayed access control (DAC). Scores are least squares means from a mixed effects model adjusting for baseline anxiety and time (piecewise acute and follow-up phases). GAD-7: Generalized Anxiety Disorder-7.

In the HLM with time treated categorically, no significant group differences were evident across the first 6 weeks (range of *Ps=*.10 to .96), but a nonsignificant trend emerged by week 7 (*P*=.08), and by weeks 8 and 9, CBI-VR showed significantly lower anxiety symptom scores compared with the other conditions (week 8: *P*=.01; week 9: *P*=.02). Similar to the PHQ-9 scores, mean GAD-7 scores remained broadly stable across conditions across the follow-up period. In the VR condition, there was a small numerical increase in GAD-7 scores at the 6-month follow-up assessment relative to the postintervention assessment; however, scores at every follow-up timepoint remained lower than those in the delayed access and CBI-FS conditions. Figure 3 shows the estimated least squares mean trajectories; slice tests and pairwise comparisons are reported in Multimedia Appendix 1.

#### Intervention Effects on Quality of Life

WHOQOL-BREF was assessed at baseline and at postintervention. Each WHOQOL-BREF subscale score was examined in separate HLMs. Across models, all of the subscale scores changed significantly from baseline to postintervention (*P*<.001). The rate of improvement differed significantly among conditions for the general quality-of-life subscale (time × condition interaction: *F*_2,556_=5.58; *P*=.004); participants in CBI-VR reported significantly greater increases in general quality-of-life scores compared with DAC (*β*=−1.02, 95% CI −1.63 to −0.40; SE=0.31; *P*=.001), but the difference between CBI-VR and CBI-FS was not significant (*β*=−.33, 95% CI −0.96 to 0.31; SE=0.32; *P*=.31). The rate of improvement also significantly differed among conditions for the psychological subscale (time × condition interaction: *F*_2,557_=11.25; *P*<.001); participants in CBI-VR reported significantly greater increases in psychological subscale scores compared with CBI-FS (*β*=−.65, 95% CI −1.08 to −0.21; SE=0.22; *P*=.004) and DAC (*β*=−1.01, 95% CI −1.44 to −0.59; SE=0.22; *P*<.001). However, the rate of improvement among conditions (time × condition interactions) did not significantly differ for the general health (*F*_2,556_=2.80; *P*=.06), physical (*F*_2,557_=1.34; *P*=.26), social (*F*_2,557_=1.99; *P*=.14), or environmental subscales (*F*_2,557_=0.61; *P*=.54).

**Figure 4.**
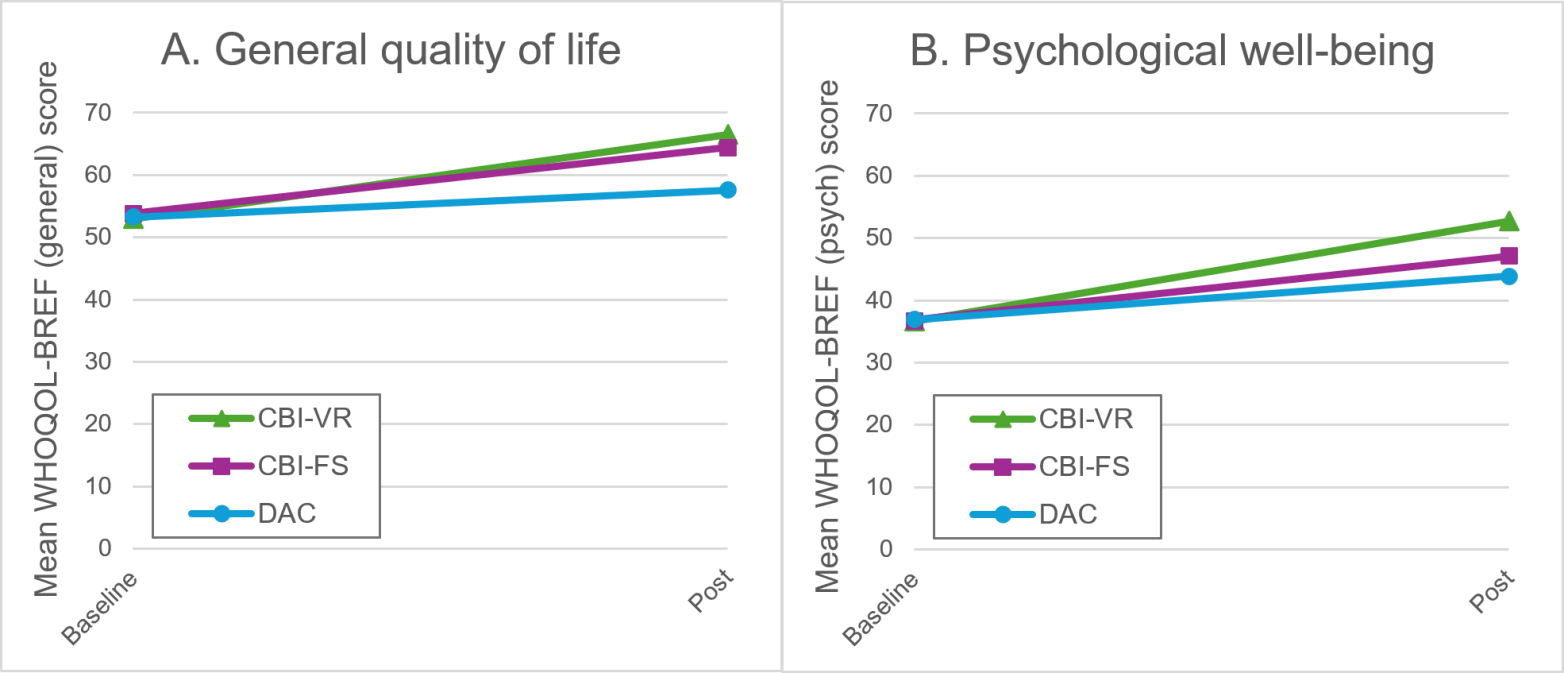
Adjusted mean general quality-of-life and psychological well-being scores over time by condition from pre- to post-intervention period in a randomized controlled trial of cognitive behavioral immersion (CBI) among adults with elevated depression symptoms in the United States. CBI accessed via a virtual reality headset (CBI-VR) or a flat-screen device (CBI-FS) and compared with a delayed access control (DAC). Scores are least squares means from a mixed-effects model adjusting for time. WHOQOL-BREF: World Health Organization Quality of Life Brief measure.

We ran HLMs treating time as a categorical variable to predict the WHOQOL-BREF subscale scores that differed between conditions in the prior models. The HLM examining WHOQOL-BREF general quality-of-life scores found no significant group differences at baseline (*F*_2, 556_=0.11; *P*=.89), but significant differences by week 9 in general quality-of-life scores among conditions (*F*_2, 556_=10.62; *P*<.001) favoring both the CBI-VR and CBI-FS conditions over DAC (*P*<.001), which did not differ from one another (*P*=.34). A parallel HLM predicting WHOQOL-BREF psychological well-being scores also found no significant group differences at baseline for this subscale (*F*_2,557_=0.02; *P*=.98), but significant differences among conditions emerged by week 9 (*F*_2,557_=19.67; *P*<.001) favoring CBI-VR over both CBI-FS and DAC (*P*<.001). Figure 4 shows the estimated least squares mean trajectories of WHOQOL-BREF general quality-of-life and psychological well-being scores; slice tests and pairwise comparisons are reported in Multimedia Appendix 1.

### Ancillary Analyses

#### Environmental Presence as a Mediator of Intervention Outcomes

Presence variables were assessed at post intervention. CBI-VR participants rated environmental presence with a mean of 18.97 (SD 4.45; range 5‐25), while CBI-FS participants rated it with a mean of 14.99 (SD 5.48; range 5‐25). A post hoc model was fit examining environmental presence as a mediator of the effect of condition (CBI-VR vs CBI-FS) on postintervention depressive symptoms while controlling for baseline depression symptoms. In this model, a significant indirect effect of environmental presence was observed (ab=−0.85, 95% CI −1.71 to −0.15; SE=0.40). Specifically, CBI-VR was associated with higher environmental presence (*a*=3.97, 95% CI 2.38 to 5.57; SE=0.81; *P*<.001), which in turn was associated with lower postintervention depressive symptoms (b=−0.21, 95% CI −0.40 to −0.03; SE=0.09; *P*=.02). Total (c=−1.06, 95% CI −2.92 to 0.80; SE=0.94; *P*=.26) and direct effects (c*’*=−0.21, 95% CI −2.19 to 1.76; SE=1.00; *P*=.83) were not significant.

#### Social Presence as a Mediator of Intervention Outcomes

CBI-VR participants rated social presence with a mean of 16.45 (SD 4.02; range 3‐21), while CBI-FS participants rated it with a mean of 15.52 (SD 3.85; range 3‐21). In a parallel post hoc model examining social presence as a mediator of condition effect, social presence did not significantly mediate the relationship between condition and depressive symptoms (ab=−0.31, 95% CI −0.83 to 0.14; SE=0.24). While higher social presence was associated with lower depressive symptoms (*b*=−0.34, 95% CI −0.56 to −0.11; SE=0.12; *P*<.01), condition did not significantly predict social presence scores (*a*=0.92, 95% CI −0.32 to 2.17; SE=0.63; *P*=.14). Total (*c*=−1.12, 95% CI −2.96 to 0.72; SE=0.93; *P*=.23) and direct effects (*c’*=−0.81, 95% CI −2.62 to 1.00; SE=0.91; *P*=.38) remained nonsignificant.

#### Pattern of Missing Data

Little MCAR test was significant (*χ*²_754_=852.40; *P*=.01), indicating that data were not missing completely at random. Further inspection of the data showed that missingness generally increased over time (refer to Multimedia Appendix 1) and appeared to be driven primarily by individuals who dropped out of the intervention and stopped completing surveys. First, we evaluated whether those who did and did not drop out varied on baseline characteristics including age, sex, race, ethnicity, and education and found no significant differences (range of *Ps*=.16 to .71).

We then ran a pattern-mixture model to examine whether symptom trajectories differed by dropout status. Specifically, we reestimated the primary HLM for PHQ-9 scores including dropout status and its interactions with time, condition, and time × condition. Dropout status did not significantly interact with time (*F*_1,606_=1.96; *P*=.16), condition (*F*_1,444_=0.54; *P*=.46), or time × condition (*F*_1,606_=0.04; *P*=.84). These findings suggest that the intervention effects were consistent across missing data patterns.

#### Coach Effects as a Covariate

We explored the potential confounding role of CBI coaches in the dataset by first examining whether GSRS scores (a proxy for session quality) varied by coach. To do so, we fit a linear mixed-effects model with participant as a random effect to test whether mean GSRS scores were associated with coach assignment. GSRS scores differed significantly by coach (*F*_7,789_=2.72; *P*=.009), suggesting that session quality ratings differed by coach. Therefore, we reestimated our primary HLM examining depression symptom slopes with the inclusion of coach as a covariate. Results did not change in direction or significance after controlling for the effect of coach.

## Discussion

### Principal Findings

The primary objective of this randomized controlled trial was to evaluate whether a coach-led, group-based cognitive behavioral skills program delivered in a shared digital environment could reduce depression and anxiety symptoms and improve quality of life, and whether immersive VR delivery conferred additional benefit over flat-screen delivery. Overall, findings supported our hypothesis that participants receiving the intervention via VR would show greater improvements in depression, anxiety, and quality of life compared to the same intervention accessed via flat-screen devices and a DAC group, with gains largely maintained over follow-up. In addition, participants receiving the same intervention via flat-screen devices did not significantly differ from the control on depression or anxiety outcomes, although they demonstrated improved general quality of life relative to the control. A further exploratory aim was to examine whether sense of presence mediated intervention response. Physical presence within the virtual environment, but not social presence, was associated with reductions in depression symptoms in the VR intervention condition.

### Interpretation

The observed symptom improvements and maintenance of gains over follow-up demonstrated in this study among participants in the CBI-VR group suggest that immersive, socially interactive digital interventions may represent a promising avenue for expanding access to behavioral support, though their effects may depend in part on the level of immersion afforded by the delivery format. This study contributes to the growing literature on digital CBT-based interventions [43,44] by demonstrating the potential clinical utility of delivering a synchronous, coach-led, group CBT skills program within a shared virtual environment. Prior research on digital CBT has largely focused on asynchronous web-based programs, self-guided mobile applications, or therapist-guided telehealth approaches [43,44]. In contrast, participants in the current study interacted with peers and coaches as avatars in shared 3D environments in real time to learn and practice CBT-based techniques [31]. This synchronous format was designed to preserve the interpersonal facets such as the therapeutic alliance, which are often considered central to producing change in traditional face-to-face therapies [45] and are gaining recent support as contributors to outcomes even in internet-based interventions [46]. The observed symptom improvements and maintenance of gains over the follow-up period suggest that immersive, socially interactive digital interventions may represent a promising avenue for expanding access to mental health interventions that provide more than short-term symptom relief by supporting the sustainment of gains after the active intervention period.

A major strength of this study was the direct comparison of immersive VR and flat-screen access to an otherwise identical intervention. The CBI program was structurally and procedurally identical across conditions; in fact, VR and flat-screen participants attended the same group sessions led by the same coaches [31]. Because participants across conditions attended the same sessions with the same coaches and content, the observed differences between groups are less likely to reflect intervention content or coach effects. Participants who accessed the intervention through immersive VR experienced greater and faster symptom improvements than those accessing the intervention via flat-screen devices, suggesting that immersive delivery may enhance engagement with or responsiveness to digital CBT-based interventions. These findings align with recent research suggesting that immersive VR may be associated with greater engagement, understanding, and learning in educational settings [eg, 47-49]. However, because the flat-screen condition did not significantly differ from the DAC, future research is needed to determine whether these effects reflect advantages specific to immersive delivery, novelty effects associated with first-time VR exposure [50], or interactions between VR and the intervention itself.

Exploratory mediation analyses suggested that environmental presence (ie, the feeling of physically inhabiting the virtual environment [21]) may represent one pathway through which immersive VR delivery contributes to symptom improvement [40]. In contrast, social presence (ie, the sense of feeling connected to others in the virtual environment [22,23]) did not significantly mediate outcomes. Because these analyses were exploratory and temporal precedence was not established between the purported mediator and outcome variables [51], this pattern of findings is broadly consistent with viewpoints proposing that immersive VR enhances physical immersion and experiential engagement beyond interpersonal connectedness alone [26,52]. Future research should measure sense of presence repeatedly across sessions, which would help establish proper temporal ordering of putative mediators and outcomes and allow for testing of whether changes in environmental presence truly precede changes in symptoms.

### Limitations

Several limitations should be noted. First, although participants reported clinically elevated depressive symptoms, formal diagnostic interviews were not conducted, and findings may not generalize to populations meeting diagnostic criteria for depression. Second, while VR hardware is becoming increasingly accessible and adopted by health care settings [53], it still presents logistical and cost barriers for some users [54]. Third, temporal precedence was not established between presence measures and outcome variables examined in the exploratory mediation models; therefore, reverse causality cannot be ruled out [51]. Links between presence and symptom improvement require further longitudinal investigation in experimental designs. Fourth, concomitant care was permitted but not systematically measured, which limits our ability to assess whether changes in treatment unrelated to the study contributed to observed symptom change across conditions. Finally, although coaches received structured training, manuals, and checklists as well as weekly supervision, formal observer-rated fidelity checks were not conducted, which limits our ability to verify intervention adherence and competence across coaches and sessions. Session quality was assessed only via participant-reported rating scales.

### Conclusions

Together, these findings support the potential of immersive VR as a delivery modality for synchronous, coach-led CBT-based group interventions targeting depression and anxiety symptoms. If replicated, results suggest that shared virtual environments, particularly when accessed via immersive formats, may help preserve relational and experiential aspects of behavioral interventions while increasing scalability and accessibility. This may be especially relevant for individuals who face barriers to in-person care but may benefit more from structured, synchronous, coach-led interventions than unguided digital tools, which may not retain the same aspects. However, the ability to scale these interventions will also depend on practical factors such as headset availability and price, comfort with novel technology, and the quality of technical support. We encourage future research to clarify mechanisms of change, evaluate long-term implementation and cost-effectiveness outcomes, and determine whether immersive delivery provides advantages beyond novelty or engagement effects alone.

## Supplementary material

10.2196/92347 Multimedia Appendix 1 Additional tables.

10.2196/92347 Multimedia Appendix 2 Letter regarding institutional review board approval.

10.2196/92347 Checklist 1 CONSORT-EHEALTH checklist.

10.2196/92347 Checklist 2 CONSORT 2025 Expanded and Abstract Extension checklists.

## Abbreviations

C-SSRS: Columbia-Suicide Severity Rating Scale
CBI: cognitive behavioral immersion
CBI-FS: cognitive behavioral immersion accessed via a flat-screen device
CBI-VR: cognitive behavioral immersion accessed via a virtual reality headset
CBT: cognitive behavioral therapy
CONSORT: Consolidated Standards of Reporting Trials
DAC: delayed access control
E-HEALTH: Consolidated Standards of Reporting Trials of Electronic and Mobile Health Applications and Online Telehealth
GAD-7: Generalized Anxiety Disorder-7
GSRS: Group Session Rating Scale
HLM: hierarchical linear model
PHQ-9: Patient Health Questionnaire-9
USC: University of Southern California
VR: virtual reality
WHOQOL-BREF: World Health Organization Quality of Life Brief
