# Supplementary material for: Cognitive Behavioral Immersion for Depression: Randomized Controlled Trial Comparing Virtual Reality and Flat-Screen Delivery
Source: J Med Internet Res. 2026 Jul 15;28:e92347. doi: 10.2196/92347 (PMC13373704; doi:10.2196/92347)
Supplement: Multimedia Appendix 1 [file jmir-v28-e92347-s001.docx]

Number of Surveys Missing per Assessment Point Separated by Condition

| **Assessment** | **CBI-VR** | | **CBI-FS** | | **DAC** | | **All conditions** | |
| --- | --- | --- | --- | --- | --- | --- | --- | --- |
|  | *%* | *n/102* | *%* | *n/102* | *%* | *n/102* | *%* | *n/306* |
| Baseline | 0 | 0 | 0 | 0 | 0 | 0 | 0 | 0 |
| Week 1 | 9.8 | 10 | 19.61 | 20 | 0 | 0 | 9.80 | 30 |
| Week 2 | 13.73 | 14 | 21.57 | 22 | 1.96 | 2 | 12.42 | 38 |
| Week 3 | 17.65 | 18 | 21.57 | 22 | 1.96 | 2 | 13.73 | 42 |
| Week 4 | 19.61 | 20 | 25.49 | 26 | 2.94 | 3 | 16.01 | 49 |
| Week 5 | 19.61 | 20 | 24.51 | 25 | 3.92 | 4 | 16.01 | 49 |
| Week 6 | 25.49 | 26 | 26.47 | 27 | 2.94 | 3 | 18.30 | 56 |
| Week 7 | 23.53 | 24 | 29.41 | 30 | 1.96 | 2 | 18.30 | 56 |
| Week 8 | 24.51 | 25 | 24.51 | 25 | 1.96 | 2 | 16.99 | 52 |
| Post | 20.59 | 21 | 24.51 | 25 | 1.96 | 2 | 15.69 | 48 |
| Month 1 | 28.43 | 29 | 29.41 | 30 | 5.88 | 6 | 21.24 | 65 |
| Month 2 | 30.39 | 31 | 30.39 | 31 | 6.86 | 7 | 22.55 | 69 |
| Month 3 | 32.35 | 33 | 32.35 | 33 | 4.9 | 5 | 23.20 | 71 |
| Month 4 | 35.29 | 36 | 35.29 | 36 | 8.82 | 9 | 26.47 | 81 |
| Month 5 | 34.31 | 35 | 38.24 | 39 | 14.71 | 15 | 29.08 | 89 |
| Month 6 | 35.29 | 36 | 35.29 | 36 | 13.73 | 14 | 28.10 | 86 |
| All assessments | 23.16 | 378/1632 | 26.16 | 427/1632 | 4.66 | 76/1632 | 17.99 | 881/4896 |

Slice Tests of the Condition × Time Interaction on Depression Scores at Each Assessment Point

| **Effect** | ***F*** | ***p*** |
| --- | --- | --- |
| Condition*Week 1 | 0.16 | .85 |
| Condition*Week 2 | 0.05 | .95 |
| Condition*Week 3 | 0.51 | .60 |
| Condition*Week 4 | 2.11 | .12 |
| Condition*Week 5 | 2.17 | .11 |
| Condition*Week 6 | 1.45 | .24 |
| Condition*Week 7 | 2.53 | .08 |
| Condition*Week 8 | 4.38 | .01 |
| Condition*Post | 4.80 | .01 |
| Condition*Month 1 | 2.59 | .08 |
| Condition*Month 2 | 1.81 | .16 |
| Condition*Month 3 | 0.78 | .46 |
| Condition*Month 4 | 3.14 | .04 |
| Condition*Month 5 | 2.90 | .06 |
| Condition*Month 6 | 1.76 | .17 |

Pairwise Differences in Least Squares Mean Depression Scores Between Conditions

| **Assessment** | **Conditions** | | ***Estimate*** | ***SE*** | ***t*** | ***p*** | ***95% CI*** | |
| --- | --- | --- | --- | --- | --- | --- | --- | --- |
| Week 1 | CBI-FS | DAC | 0.01 | 0.75 | 0.01 | .99 | -1.47 | 1.49 |
| Week 1 | CBI-FS | CBI-VR | -0.36 | 0.76 | -0.47 | .64 | -1.87 | 1.14 |
| Week 1 | DAC | CBI-VR | -0.37 | 0.73 | -0.51 | .61 | -1.80 | 1.06 |
| Week 2 | CBI-FS | DAC | -0.07 | 0.76 | -0.09 | .93 | -1.55 | 1.42 |
| Week 2 | CBI-FS | CBI-VR | 0.16 | 0.77 | 0.21 | .84 | -1.36 | 1.68 |
| Week 2 | DAC | CBI-VR | 0.23 | 0.74 | 0.31 | .76 | -1.22 | 1.67 |
| Week 3 | CBI-FS | DAC | -0.77 | 0.76 | -1.01 | .31 | -2.26 | 0.72 |
| Week 3 | CBI-FS | CBI-VR | -0.40 | 0.78 | -0.51 | .61 | -1.93 | 1.13 |
| Week 3 | DAC | CBI-VR | 0.37 | 0.74 | 0.50 | .62 | -1.09 | 1.82 |
| Week 4 | CBI-FS | DAC | -0.35 | 0.76 | -0.46 | .65 | -1.85 | 1.15 |
| Week 4 | CBI-FS | CBI-VR | 1.13 | 0.79 | 1.44 | .15 | -0.41 | 2.68 |
| Week 4 | DAC | CBI-VR | 1.48 | 0.74 | 1.99 | .05 | 0.02 | 2.94 |
| Week 5 | CBI-FS | DAC | -0.43 | 0.76 | -0.56 | .58 | -1.92 | 1.07 |
| Week 5 | CBI-FS | CBI-VR | 1.10 | 0.79 | 1.40 | .16 | -0.45 | 2.64 |
| Week 5 | DAC | CBI-VR | 1.52 | 0.75 | 2.04 | .04 | 0.06 | 2.98 |
| Week 6 | CBI-FS | DAC | -0.28 | 0.77 | -0.36 | .72 | -1.78 | 1.23 |
| Week 6 | CBI-FS | CBI-VR | 0.96 | 0.80 | 1.21 | .23 | -0.60 | 2.52 |
| Week 6 | DAC | CBI-VR | 1.24 | 0.75 | 1.65 | .10 | -0.24 | 2.72 |
| Week 7 | CBI-FS | DAC | -0.94 | 0.77 | -1.22 | .22 | -2.45 | 0.57 |
| Week 7 | CBI-FS | CBI-VR | 0.74 | 0.80 | 0.93 | .35 | -0.82 | 2.30 |
| Week 7 | DAC | CBI-VR | 1.68 | 0.75 | 2.24 | .03 | 0.21 | 3.15 |
| Week 8 | CBI-FS | DAC | -0.82 | 0.76 | -1.08 | .28 | -2.32 | 0.67 |
| Week 8 | CBI-FS | CBI-VR | 1.39 | 0.79 | 1.75 | .08 | -0.17 | 2.94 |
| Week 8 | DAC | CBI-VR | 2.21 | 0.75 | 2.95 | .003 | 0.74 | 3.68 |
| Post | CBI-FS | DAC | -1.14 | 0.76 | -1.50 | .13 | -2.64 | 0.36 |
| Post | CBI-FS | CBI-VR | 1.17 | 0.79 | 1.48 | .14 | -0.38 | 2.71 |
| Post | DAC | CBI-VR | 2.31 | 0.75 | 3.10 | <.001 | 0.85 | 3.77 |
| Month 1 | CBI-FS | DAC | -0.38 | 0.77 | -0.49 | .63 | -1.89 | 1.14 |
| Month 1 | CBI-FS | CBI-VR | 1.30 | 0.80 | 1.62 | .11 | -0.28 | 2.87 |
| Month 1 | DAC | CBI-VR | 1.68 | 0.76 | 2.21 | .03 | 0.18 | 3.17 |
| Month 2 | CBI-FS | DAC | -0.62 | 0.77 | -0.80 | .42 | -2.14 | 0.90 |
| Month 2 | CBI-FS | CBI-VR | 0.83 | 0.81 | 1.03 | .30 | -0.75 | 2.41 |
| Month 2 | DAC | CBI-VR | 1.45 | 0.76 | 1.90 | .06 | -0.05 | 2.95 |
| Month 3 | CBI-FS | DAC | -0.27 | 0.78 | -0.35 | .73 | -1.79 | 1.26 |
| Month 3 | CBI-FS | CBI-VR | 0.67 | 0.81 | 0.82 | .41 | -0.93 | 2.27 |
| Month 3 | DAC | CBI-VR | 0.94 | 0.76 | 1.23 | .22 | -0.56 | 2.44 |
| Month 4 | CBI-FS | DAC | -0.77 | 0.78 | -0.98 | .33 | -2.30 | 0.77 |
| Month 4 | CBI-FS | CBI-VR | 1.17 | 0.82 | 1.42 | .16 | -0.45 | 2.78 |
| Month 4 | DAC | CBI-VR | 1.93 | 0.77 | 2.50 | .01 | 0.42 | 3.45 |
| Month 5 | CBI-FS | DAC | -1.02 | 0.79 | -1.28 | .20 | -2.58 | 0.54 |
| Month 5 | CBI-FS | CBI-VR | 0.84 | 0.83 | 1.02 | .31 | -0.78 | 2.46 |
| Month 5 | DAC | CBI-VR | 1.86 | 0.78 | 2.40 | .02 | 0.34 | 3.39 |
| Month 6 | CBI-FS | DAC | -1.47 | 0.79 | -1.87 | .06 | -3.02 | 0.08 |
| Month 6 | CBI-FS | CBI-VR | -0.70 | 0.82 | -0.86 | .39 | -2.32 | 0.91 |
| Month 6 | DAC | CBI-VR | 0.77 | 0.78 | 0.99 | .32 | -0.76 | 2.29 |

Slice Tests of the Condition × Time Interaction on Anxiety Scores at Each Assessment Point

| **Effect** | ***F*** | ***p*** |
| --- | --- | --- |
| Condition*Week 1 | 0.05 | .96 |
| Condition*Week 2 | 0.88 | .42 |
| Condition*Week 3 | 1.50 | .22 |
| Condition*Week 4 | 1.92 | .15 |
| Condition*Week 5 | 1.90 | .15 |
| Condition*Week 6 | 2.30 | .10 |
| Condition*Week 7 | 2.59 | .08 |
| Condition*Week 8 | 5.22 | .01 |
| Condition*Post | 4.10 | .02 |
| Condition*Month 1 | 2.16 | .12 |
| Condition*Month 2 | 2.20 | .11 |
| Condition*Month 3 | 2.58 | .08 |
| Condition*Month 4 | 4.81 | .01 |
| Condition*Month 5 | 5.47 | .004 |
| Condition*Month 6 | 4.50 | .01 |

Pairwise Differences in Least Squares Mean Anxiety Scores Between Conditions

| **Assessment** | **Conditions** | | ***Estimate*** | ***SE*** | ***t*** | ***p*** | ***95% CI*** | |
| --- | --- | --- | --- | --- | --- | --- | --- | --- |
| Week 1 | CBI-FS | DAC | -0.12 | 0.71 | -0.17 | .86 | -1.52 | 1.27 |
| Week 1 | CBI-FS | CBI-VR | -0.22 | 0.72 | -0.30 | .76 | -1.64 | 1.20 |
| Week 1 | DAC | CBI-VR | -0.09 | 0.69 | -0.14 | .89 | -1.45 | 1.26 |
| Week 2 | CBI-FS | DAC | -0.81 | 0.72 | -1.12 | .26 | -2.21 | 0.60 |
| Week 2 | CBI-FS | CBI-VR | -0.01 | 0.73 | -0.02 | .99 | -1.45 | 1.43 |
| Week 2 | DAC | CBI-VR | 0.79 | 0.70 | 1.14 | .25 | -0.57 | 2.16 |
| Week 3 | CBI-FS | DAC | -1.01 | 0.72 | -1.42 | .16 | -2.42 | 0.39 |
| Week 3 | CBI-FS | CBI-VR | 0.06 | 0.74 | 0.08 | .93 | -1.38 | 1.51 |
| Week 3 | DAC | CBI-VR | 1.08 | 0.70 | 1.54 | .12 | -0.30 | 2.45 |
| Week 4 | CBI-FS | DAC | -0.21 | 0.72 | -0.29 | .77 | -1.63 | 1.21 |
| Week 4 | CBI-FS | CBI-VR | 1.09 | 0.74 | 1.47 | .14 | -0.36 | 2.55 |
| Week 4 | DAC | CBI-VR | 1.30 | 0.70 | 1.86 | .06 | -0.07 | 2.68 |
| Week 5 | CBI-FS | DAC | -0.17 | 0.72 | -0.24 | .81 | -1.59 | 1.24 |
| Week 5 | CBI-FS | CBI-VR | 1.11 | 0.74 | 1.50 | .13 | -0.34 | 2.57 |
| Week 5 | DAC | CBI-VR | 1.29 | 0.70 | 1.83 | .07 | -0.09 | 2.67 |
| Week 6 | CBI-FS | DAC | -0.23 | 0.72 | -0.31 | .75 | -1.65 | 1.19 |
| Week 6 | CBI-FS | CBI-VR | 1.22 | 0.75 | 1.62 | .11 | -0.26 | 2.69 |
| Week 6 | DAC | CBI-VR | 1.44 | 0.71 | 2.03 | .04 | 0.05 | 2.84 |
| Week 7 | CBI-FS | DAC | -0.56 | 0.73 | -0.77 | .44 | -1.99 | 0.87 |
| Week 7 | CBI-FS | CBI-VR | 1.04 | 0.75 | 1.37 | .17 | -0.44 | 2.52 |
| Week 7 | DAC | CBI-VR | 1.60 | 0.71 | 2.26 | .02 | 0.21 | 2.98 |
| Week 8 | CBI-FS | DAC | -0.76 | 0.72 | -1.05 | .29 | -2.17 | 0.66 |
| Week 8 | CBI-FS | CBI-VR | 1.51 | 0.75 | 2.02 | .04 | 0.04 | 2.98 |
| Week 8 | DAC | CBI-VR | 2.27 | 0.71 | 3.20 | .001 | 0.88 | 3.66 |
| Post | CBI-FS | DAC | -0.49 | 0.72 | -0.69 | .49 | -1.91 | 0.92 |
| Post | CBI-FS | CBI-VR | 1.47 | 0.74 | 1.97 | .05 | 0.01 | 2.93 |
| Post | DAC | CBI-VR | 1.96 | 0.70 | 2.79 | .01 | 0.58 | 3.34 |
| Month 1 | CBI-FS | DAC | 0.42 | 0.73 | 0.58 | .56 | -1.01 | 1.86 |
| Month 1 | CBI-FS | CBI-VR | 1.51 | 0.76 | 2.00 | .05 | 0.03 | 3.00 |
| Month 1 | DAC | CBI-VR | 1.09 | 0.72 | 1.52 | .13 | -0.32 | 2.50 |
| Month 2 | CBI-FS | DAC | -0.83 | 0.73 | -1.13 | .26 | -2.27 | 0.61 |
| Month 2 | CBI-FS | CBI-VR | 0.68 | 0.76 | 0.89 | .38 | -0.82 | 2.17 |
| Month 2 | DAC | CBI-VR | 1.50 | 0.72 | 2.09 | .04 | 0.09 | 2.92 |
| Month 3 | CBI-FS | DAC | -0.27 | 0.73 | -0.37 | .71 | -1.70 | 1.17 |
| Month 3 | CBI-FS | CBI-VR | 1.30 | 0.77 | 1.69 | .09 | -0.21 | 2.81 |
| Month 3 | DAC | CBI-VR | 1.57 | 0.72 | 2.17 | .03 | 0.15 | 2.99 |
| Month 4 | CBI-FS | DAC | -0.17 | 0.74 | -0.22 | .82 | -1.62 | 1.29 |
| Month 4 | CBI-FS | CBI-VR | 1.93 | 0.78 | 2.49 | .01 | 0.41 | 3.45 |
| Month 4 | DAC | CBI-VR | 2.09 | 0.73 | 2.87 | <.001 | 0.66 | 3.53 |
| Month 5 | CBI-FS | DAC | -0.43 | 0.75 | -0.57 | .57 | -1.90 | 1.04 |
| Month 5 | CBI-FS | CBI-VR | 1.89 | 0.78 | 2.43 | .02 | 0.36 | 3.42 |
| Month 5 | DAC | CBI-VR | 2.32 | 0.73 | 3.17 | <.001 | 0.88 | 3.76 |
| Month 6 | CBI-FS | DAC | -1.50 | 0.74 | -2.02 | .04 | -2.97 | -0.04 |
| Month 6 | CBI-FS | CBI-VR | 0.61 | 0.78 | 0.79 | .43 | -0.91 | 2.14 |
| Month 6 | DAC | CBI-VR | 2.12 | 0.73 | 2.89 | .004 | 0.68 | 3.56 |

Slice Tests of the Condition × Time Interaction on General Quality of Life Scores at Each Assessment Point

| **Effect** | ***F*** | ***p*** |
| --- | --- | --- |
| Condition*Baseline | 0.11 | .89 |
| Condition*Post | 10.62 | <.001 |

Pairwise Differences in Least Squares Mean General Quality of Life Scores Between Conditions

| **Assessment** | **Conditions** | | ***Estimate*** | ***SE*** | ***t*** | ***p*** | ***95% CI*** | |
| --- | --- | --- | --- | --- | --- | --- | --- | --- |
| Baseline | DAC | VR | 0.01 | 0.08 | 0.11 | .91 | -0.14 | 0.16 |
| Baseline | FS | DAC | 0.03 | 0.08 | 0.34 | .73 | -0.12 | 0.18 |
| Baseline | FS | VR | 0.04 | 0.08 | 0.46 | .65 | -0.12 | 0.19 |
| Post | FS | DAC | 0.27 | 0.08 | 3.29 | <.001 | 0.11 | 0.44 |
| Post | FS | VR | -0.08 | 0.09 | -0.95 | .34 | -0.25 | 0.09 |
| Post | DAC | VR | -0.36 | 0.08 | -4.35 | <.001 | -0.52 | -0.20 |

Slice Tests of the Condition × Time Interaction on Psychological Wellbeing Scores at Each Assessment Point

| **Effect** | ***F*** | ***p*** |
| --- | --- | --- |
| Condition*Baseline | 0.02 | .98 |
| Condition*Post | 19.67 | <.001 |

Pairwise Differences in Least Squares Mean Psychological Wellbeing Scores Between Conditions

| **Assessment** | **Conditions** | | ***Estimate*** | ***SE*** | ***t*** | ***p*** | ***95% CI*** | |
| --- | --- | --- | --- | --- | --- | --- | --- | --- |
| Baseline | DAC | VR | 0.25 | 1.33 | 0.19 | .85 | -2.36 | 2.86 |
| Baseline | FS | DAC | -0.04 | 1.33 | -0.03 | .98 | -2.64 | 2.57 |
| Baseline | FS | VR | 0.21 | 1.33 | 0.16 | .87 | -2.39 | 2.82 |
| Post | FS | DAC | 3.28 | 1.44 | 2.28 | .02 | 0.46 | 6.10 |
| Post | FS | VR | -5.60 | 1.51 | -3.71 | <.001 | -8.57 | -2.64 |
| Post | DAC | VR | -8.88 | 1.42 | -6.25 | <.001 | -11.67 | -6.09 |
