# Supplementary material for: Cognitive Behavioral Immersion for Depression: Randomized Controlled Trial Comparing Virtual Reality and Flat-Screen Delivery
Source: J Med Internet Res. 2026 Jul 15;28:e92347. doi: 10.2196/92347 (PMC13373704; doi:10.2196/92347)
Supplement: Multimedia Appendix 2 [file jmir-v28-e92347-s002.pdf]

USC Research and Innovation
Human Research Protection Program

June 3, 2026

To Whom It May Concern:

This letter confirms that the methodology described in the manuscript entitled “Cognitive Behavioral Immersion for Depression: Virtual Reality Associated with Faster Symptom Reduction Than Flat-Screens in Randomized Controlled Trial" is the same as the methodology approved by the University of Southern California Institutional Review Board under Protocol # UP-23-00491 when ethics approval was issued.

Sincerely,

Maria Brown, MPH, CIP, CCRP
Reliance Lead IRB Analyst
University of Southern California
