# Supplementary material for: Cognitive Behavioral Immersion for Depression: Randomized Controlled Trial Comparing Virtual Reality and Flat-Screen Delivery
Source: J Med Internet Res. 2026 Jul 15;28:e92347. doi: 10.2196/92347 (PMC13373704; doi:10.2196/92347)
Supplement: Checklist 2 [file jmir-v28-e92347-s004.docx]

CONSORT 2025 Expanded Checklist

| **Section / Topic** | **No** | **CONSORT 2025 checklist item description** | **Reported on page no.** |
| --- | --- | --- | --- |
| **Title and abstract** | | |  |
| Title and structured abstract | 1a | Identification as a randomised trial | 1, line 3;  2, line 14 |
|  | 1b | Structured summary of the trial design, methods, results, and conclusions | 2, lines 14-47 |
| **Open science** | | |  |
| Trial registration | 2 | Name of trial registry, identifying number (with URL) and date of registration | 2, lines 49-51 |
| Protocol and statistical analysis plan | 3 | Where the trial protocol and statistical analysis plan can be accessed | 22, lines 733-735 |
| Data sharing | 4 | Where and how the individual de-identified participant data (including data dictionary), statistical code and any other materials can be accessed | 3, lines 52-54 |
| Funding and conflicts of interest | 5a | Sources of funding and other support (e.g., supply of drugs), and role of funders in the design, conduct, analysis and reporting of the trial | 3, lines 56-57 |
|  | 5b | Financial and other conflicts of interest of the manuscript authors | 3, lines 56-57; 22, lines 727-730 |
| **Introduction** | | |  |
| Background and rationale | 6 | Scientific background and rationale | 4-5, lines 62-139 |
| Objectives | 7 | Specific objectives related to benefits and harms | 5-6, lines 140-155 |
| **Methods** | | |  |
| Patient and public involvement | 8 | Details of patient or public involvement in the design, conduct and reporting of the trial | 6, lines 157-159 |
| Trial design | 9 | Description of trial design including type of trial (e.g., parallel group, crossover), allocation ratio, and framework (e.g., superiority, equivalence, non-inferiority, exploratory) | 6, lines 160-164 |
| Changes to trial protocol | 10 | Important changes to the trial after it commenced including any outcomes or analyses that were not prespecified, with reason | 6, lines 165-168 |
| Trial setting | 11 | Settings (e.g., community, hospital) and locations (e.g., countries, sites) where the trial was conducted | 6, lines 169-174 |
| Eligibility criteria | 12a | Eligibility criteria for participants | 6, lines 175-184 |
|  | 12b | If applicable, eligibility criteria for sites and for individuals delivering the interventions (e.g., surgeons, physiotherapists) | for sites, N/A; 6, lines 184-189 |
| Intervention and comparator | 13 | Intervention and comparator with sufficient details to allow replication. If relevant, where additional materials describing the intervention and comparator (e.g., intervention manual) can be accessed | 6-7, lines 190-231; 12-13, lines 400-417 |
| Outcomes | 14 | Pre-specified primary and secondary outcomes, including the specific measurement variable (e.g., systolic blood pressure), analysis metric (e.g., change from baseline, final value, time to event), method of aggregation (e.g., median, proportion), and time point for each outcome | 7-8, lines 232-260 |
| Harms | 15 | How harms were defined and assessed (e.g., systematically, non-systematically) | 8, lines 261-276 |
| Sample size | 16a | How sample size was determined, including all assumptions supporting the sample size calculation | 9, lines 286-294 |
|  | 16b | Explanation of any interim analyses and stopping guidelines | 9, lines 294-297 |
| Randomisation: |  |  |  |
| Sequence generation | 17a | Who generated the random allocation sequence and the method used | 9, lines 298-300, lines 312-313 |
|  | 17b | Type of randomisation and details of any restriction (e.g., stratification, blocking and block size) | 9, lines 300-310 |
| Allocation concealment mechanism | 18 | Mechanism used to implement the random allocation sequence (e.g., central computer/telephone; sequentially numbered, opaque, sealed containers), describing any steps to conceal the sequence until interventions were assigned | 9, lines 306-315 |
| Implementation | 19 | Whether the personnel who enrolled and those who assigned participants to the interventions had access to the random allocation sequence | 9, lines 312-315 |
| Blinding | 20a | Who was blinded after assignment to interventions (e.g., participants, care providers, outcome assessors, data analysts) | 9, lines 316-319 |
|  | 20b | If blinded, how blinding was achieved and description of the similarity of interventions | N/A (unblinded; 9, lines 316-319) |
| Statistical methods | 21a | Statistical methods used to compare groups for primary and secondary outcomes, including harms | 9-10, lines 320-357 |
|  | 21b | Definition of who is included in each analysis (e.g., all randomised participants), and in which group | 10, lines 350-354 |
|  | 21c | How missing data were handled in the analysis | 10, lines 367-352 |
|  | 21d | Methods for any additional analyses (e.g., subgroup and sensitivity analyses), distinguishing prespecified from post-hoc | 10-11, lines 359-379 |
| **Results** | | |  |
| Participant flow, including flow diagram | 22a | For each group, the numbers of participants who were randomly assigned, received intended intervention, and were analysed for the primary outcome | 11, lines 396-402; 12, Fig. 1 |
|  | 22b | For each group, losses and exclusions after randomisation, together with reasons | 12, Fig. 1 |
| Recruitment | 23a | Dates defining the periods of recruitment and follow-up for outcomes of benefits and harms | 12, lines 404-408 |
|  | 23b | If relevant, why the trial ended or was stopped | 12, lines 404-407 |
| Intervention and comparator delivery | 24a | Intervention and comparator as they were actually administered (e.g., where appropriate, who delivered the intervention/comparator, how participants adhered, whether they were delivered as intended [fidelity]) | 12-13, lines 409-427 |
|  | 24b | Concomitant care received during the trial for each group | 7, lines 212-214; 13, lines 426-427 |
| Baseline data | 25 | A table showing baseline demographic and clinical characteristics for each group | 13-14, Table 1; 13, lines 428-433 |
| Numbers analysed,  outcomes and estimation | 26 | For each primary and secondary outcome, by group:   - the number of participants included in the analysis - the number of participants with available data at the outcome time point - result for each group, and the estimated effect size and its precision (such as 95% confidence interval) - for binary outcomes, presentation of both absolute and relative effect size | 14-18, lines 442-585, Figs. 2-4; Multimedia Appendix 3 |
| Harms | 27 | All harms or unintended events in each group | 8-9, lines 278-285 |
| Ancillary analyses | 28 | Any other analyses performed, including subgroup and sensitivity analyses, distinguishing pre-specified from post-hoc | 19-20, lines 587-631 |
| **Discussion** | | |  |
| Interpretation | 29 | Interpretation consistent with results, balancing benefits and harms, and considering other relevant evidence | 20-21, lines 633-693; 22, lines 709-721 |
| Limitations | 30 | Trial limitations, addressing sources of potential bias, imprecision, generalisability, and, if relevant, multiplicity of analyses | 21-22, lines 694-708 |

*We strongly recommend reading this statement in conjunction with the CONSORT 2025 Explanation and Elaboration and/or the CONSORT 2025 Expanded Checklist for important clarifications on all the items. We also recommend reading relevant CONSORT extensions. See [www.consort-spirit.org](http://www.consort-spirit.org).

Citation: Hopewell S, Chan AW, Collins GS, Hróbjartsson A, Moher D, Schulz KF, et al. CONSORT 2025 Statement: updated guideline for reporting randomised trials. BMJ. 2025; 388:e081123. <https://dx.doi.org/10.1136/bmj-2024-081123>.

© 2025 Hopewell et al. This is an Open Access article distributed under the terms of the Creative Commons Attribution License (<https://creativecommons.org/licenses/by/4.0/>), which permits unrestricted use, distribution, and reproduction in any medium, provided the original work is properly cited.

CONSORT Extension for Abstracts Checklist

| **Item** | **Description** | **Reported on line number** |
| --- | --- | --- |
| Title | Identification of the study as randomized | 3, 14 |
| Authors * | Contact details for the corresponding author | n/a |
| Trial design | Description of the trial design (e.g. parallel, cluster, non-inferiority) | 14 |
| Methods |  |  |
| Participants | Eligibility criteria for participants and the settings where the data were collected | 17-19, 23 |
| Interventions | Interventions intended for each group | 14-16, 20-21 |
| Objective | Specific objective or hypothesis | 14-16 |
| Outcome | Clearly defined primary outcome for this report | 22 |
| Randomization | How participants were allocated to interventions | 19-20 |
| Blinding (masking) | Whether or not participants, care givers, and those assessing the outcomes were blinded to group assignment | 24 |
| Results |  |  |
| Numbers randomized | Number of participants randomized to each group | 27-28 |
| Recruitment | Trial status | 22 |
| Numbers analysed | Number of participants analysed in each group | 29-31 |
| Outcome | For the primary outcome, a result for each group and the estimated effect size and its precision | 31-35 |
| Harms | Important adverse events or side effects | 40 |
| Conclusions | General interpretation of the results | 41-47 |
| Trial registration | Registration number and name of trial register | 49-50 |
| Funding | Source of funding | 56-57 |

**this item is specific to conference abstracts*
